# Leptin-deficient mice have altered three-dimensional growth plate histomorphometry

**DOI:** 10.1186/s13098-019-0402-5

**Published:** 2019-01-24

**Authors:** Jun Hung, Layla Al-Nakkash, Tom L. Broderick, Monica Castro, Jeffrey H. Plochocki

**Affiliations:** 1 Arizona College of Osteopathic Medicine, Midwestern University, Glendale, AZ 85308 USA; 2 Department of Physiology, Midwestern University, Glendale, AZ 85308 USA; 3 Laboratory of Diabetes and Exercise Metabolism, Midwestern University, Glendale, AZ 85308 USA; 4 Department of Anatomy, Midwestern University, Glendale, AZ 85308 USA; 5 Department of Medical Education, University of Central Florida College of Medicine, 6850 Lake Nona Blvd, Orlando, FL 32827 USA

**Keywords:** Obesity, Leptin, Ob/ob mice, Bone, Cartilage, Chondrocytes

## Abstract

**Background:**

Leptin is an adipokine that regulates energy homeostasis and is also needed for normal bone growth and maintenance. Mutation in the *lep* gene, which characterizes the ob/ob mouse model, results in the development of obesity and type 2 diabetes mellitus, as well as reduced limb bone length and increased fracture risk. However, the relationship between limb bone length and growth plate cartilage structure in obese diabetic adolescents is incompletely understood. Here, we tested the hypothesis that leptin deficiency affects the microstructure of growth plate cartilage in juvenile ob/ob mice.

**Methods:**

Tibial growth plate cartilage structure was compared between lean and obese, leptin-deficient (ob/ob) female mice aged 10 weeks. We used confocal laser scanning microscopy to assess 3D histological differences in Z stacks of growth plate cartilage at 0.2 µm intervals, 80–100 µm in depth. Histomorphometric comparisons were made between juvenile lean and ob/ob mice.

**Results:**

We found obese mice have significantly reduced tibial length and growth plate height in comparison with lean mice (P < 0.05). Obese mice also have fewer chondrocyte columns in growth plate cartilage with reduced chondrocyte cell volumes relative to lean mice (P < 0.05).

**Conclusions:**

These data help explicate the relationship between growth plate cartilage structure and bone health in obese diabetic juvenile mice. Our findings suggest obesity and diabetes may adversely affect growth plate cartilage structure.

**Electronic supplementary material:**

The online version of this article (10.1186/s13098-019-0402-5) contains supplementary material, which is available to authorized users.

## Introduction

Obesity and type 2 diabetes mellitus (T2DM) are on the rise worldwide in children and adolescents [1, 2]. T2DM now accounts for 45% of all new onset diabetes cases in juveniles, up from 3% just two decades ago [1]. This is a serious health concern as T2DM adversely affects growth and development, resulting in long-term, disabling complications [1]. Once such complication is impaired bone formation and mineralization, which is associated with decelerated bone growth, increased risk of bone fracture, and delayed fracture repair [3]. Impairment of bone formation is caused, at least in part, by leptin dysregulation [3–5]. Leptin is an adipokine secreted primarily by adipocytes to regulate energy homeostasis, but it also plays a role in regulating bone metabolism [6, 7]. Ob/ob mice, which are leptin deficient due to a *lep* gene mutation, are hyperphagic and exhibit metabolic signatures consistent with the T2DM phenotype [8]. Ob/ob mice also have significantly reduce bone mineral density and shorter limb bones than age-matched wild type mice [9–11].

Longitudinal growth of long bones occurs via endochondral ossification. During this process, growth plate cartilage expands and is replaced with bone tissue. Proliferation, differentiation, and metabolic activity of chondrocytes in the growth plate are inhibited in the obese, T2DM condition [12]. Leptin-deficient mice have growth plates that are reduced in height, likely due to the downregulation of genes regulating ossification [10, 13], although the specific effects of leptin-deficiency on three-dimensional growth plate structure remain unclear. In this study, we used three dimensional histomorphometric analysis to compare long bone growth plate microstructure in lean and leptin-deficient ob/ob mice. The goal was to elucidate the effects of leptin deficiency on growth plate morphology and to improve our understanding of the relationship between growth plate structure and long bone growth in ob/ob mice.

## Materials and methods

Female obese ob/ob mice (n = 5) and lean +/+ mice (n = 5) of the strain C57Bl/6-Lep^ob^ aged 4–5 weeks were purchased for the study (Jackson Laboratory; Bar Harbor, ME, USA). Ob/ob mice in this age range demonstrate obesity and hyperglycemia, as well as reduced thickness of the growth plate [10, 11]. All animals were housed in a facility with a 12 h light/dark cycle at a temperature of 22 °C. Mice were given ad libitum access to standard rodent chow and drinking water, and were treated in accordance with the National Institutes of Health’s Guide for the Care and Use of Laboratory Animals. Use of animals was approved by the Institutional Animal Care and Use Committee at Midwestern University.

**Fig. 1:**
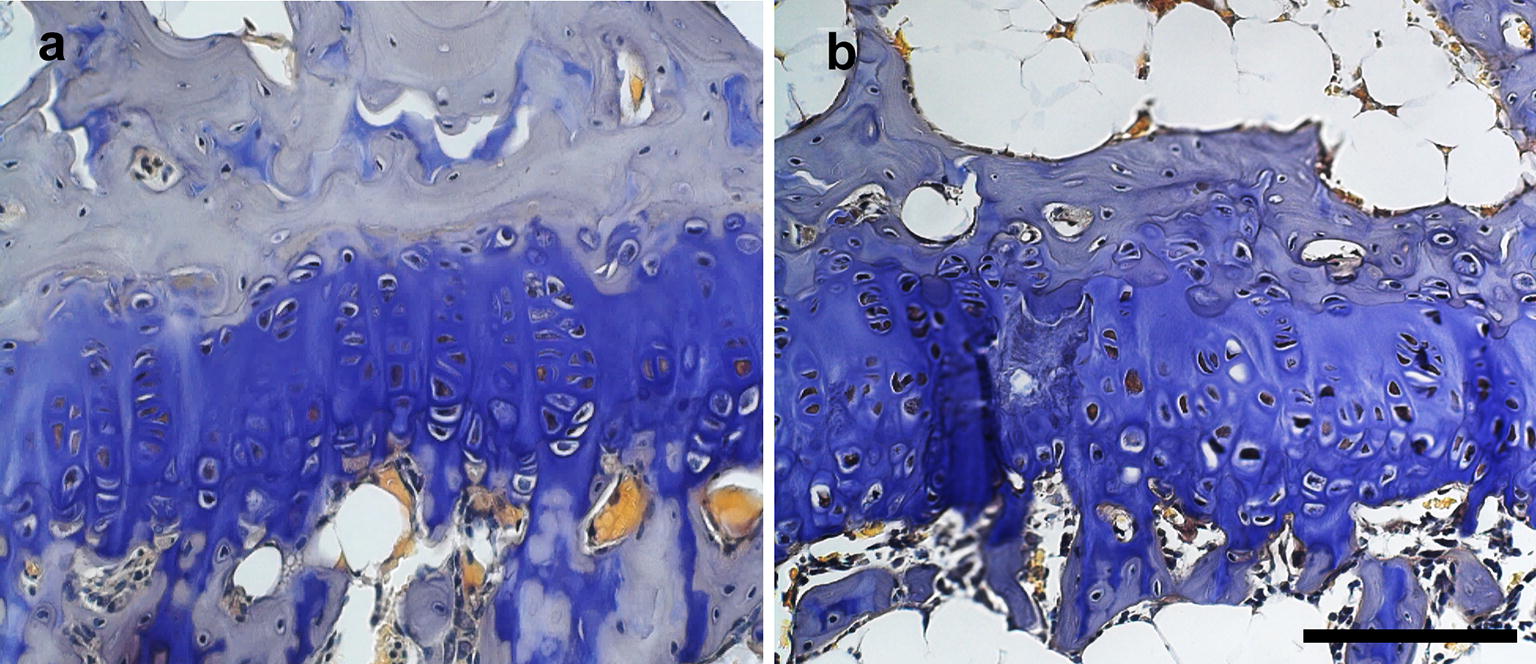
Brightfield microscopy images of growth plate cartilage in **a** lean and **b** obese mice. Chondrocytes in the growth plates of lean mice are linearly arrange while those of ob/ob mice are not. Toluidine blue stain. Scale bar is 100 µm

At 9–10 weeks of age, mice were sacrificed using compressed CO_2_ followed immediately by bilateral pneumothorax and tibias harvested for analysis. Tibias were chosen because we previously found reduced tibial length in ob/ob mice aged 6 weeks, suggesting reduced growth plate activity [11]. Length of the tibia was measured using digital calipers. Tibias were then bisected longitudinally in the sagittal plane and the medial half was prepared for imaging using confocal laser scanning microscopy [14]. Tibias were incubated in DAPI (dilution 1:800) and refractive index matching solution (RIMS) media for 48 h to reduce tissue opacity and facilitate optical imaging at greater tissue depths. Z stacks of the proximal tibia growth plate were digitally captured at 0.2 µm intervals over a range of 80–100 µm using ACS APO 40×/1.15 oil (Leica SPE confocal microscope, Leica Microsystems, Buffalo Grove, IL). Composite Z stacks in the green–blue (488 nm laser line) and yellow–green (543 nm laser line) emission spectra were formed to capture DAPI-stained nuclei and the autofluorescence of the cartilage and surrounding tissues (Fig. 1). Leica Application Suite Advanced Fluorescence software (LAS AF) algorithms were used to compose 3D images with an optical resolution in the z-axis of 0.2 µm (Leica Microsystems, v2.4.1). Image stacks were further manipulated in ImageJ and Icy ImageJ v1.6 (NIH), Icy (http://www.bioimageanlalysis.org), and 3D Visualization-Assisted Analysis software suite (Vaa3D, vaa3d.org), which were used to obtain counts of chondrocytes and chondrocyte cell columns, and to calculate the volume and surface area of each chondrocyte. Cell columns were identified from a proximal (superior) view and followed distally through the growth plate. Measuring processes were automated using the object manager in Vaa3D and were manually checked for accuracy. The mean height of the growth plate was also measured for each tibia. Data collection was blinded and conducted by a single observer.

Statistical comparisons of histomorphometric data were made using SPSS Statistics 25 (IBM, USA). Unpaired two-sampled t tests were performed with statistical significance set at *P* < 0.05. Kolmogorov–Smirnov and Levene’s tests were used to ensure assumptions of normality and equality of variances were not violated. Data are present as mean ± standard error.

## Results

Descriptive statistics for body mass and tibial dimensions are shown in Table 1. Ob/ob mice aged 5 weeks had a significantly larger mean body mass than lean mice (1.8% difference, *P* < 0.01). The difference in mean body mass between groups was significantly increased at the end of the 4-week study (5.8% difference, *P* < 0.01). Tibias of ob/ob mice were significantly shorter than those of lean controls (*P* < 0.05), with reduce mean growth plate height (*P* < 0.05).

**Table 1:** Comparisons of body mass and tibial dimensions in lean (+/+) and obese (ob/ob) mice

	Lean (+/+)	Obese (ob/ob)	*P*
Body mass at 5 weeks (g)	21.7 ± 0.64	22.1 ± 0.85	0.01
Body mass at 10 weeks (g)	23.4 ± 0.34	24.8 ± 0.95	0.01
Tibia length (mm)	17.9 ± 0.11	17.2 ± 0.61	0.02
Growth plate height (mm)	1.58 ± 0.12	0.84 ± 0.22	0.01
Chondrocyte column count	53.9 ± 8.2	43.6 ± 2.7	0.02

**Fig. 2:**
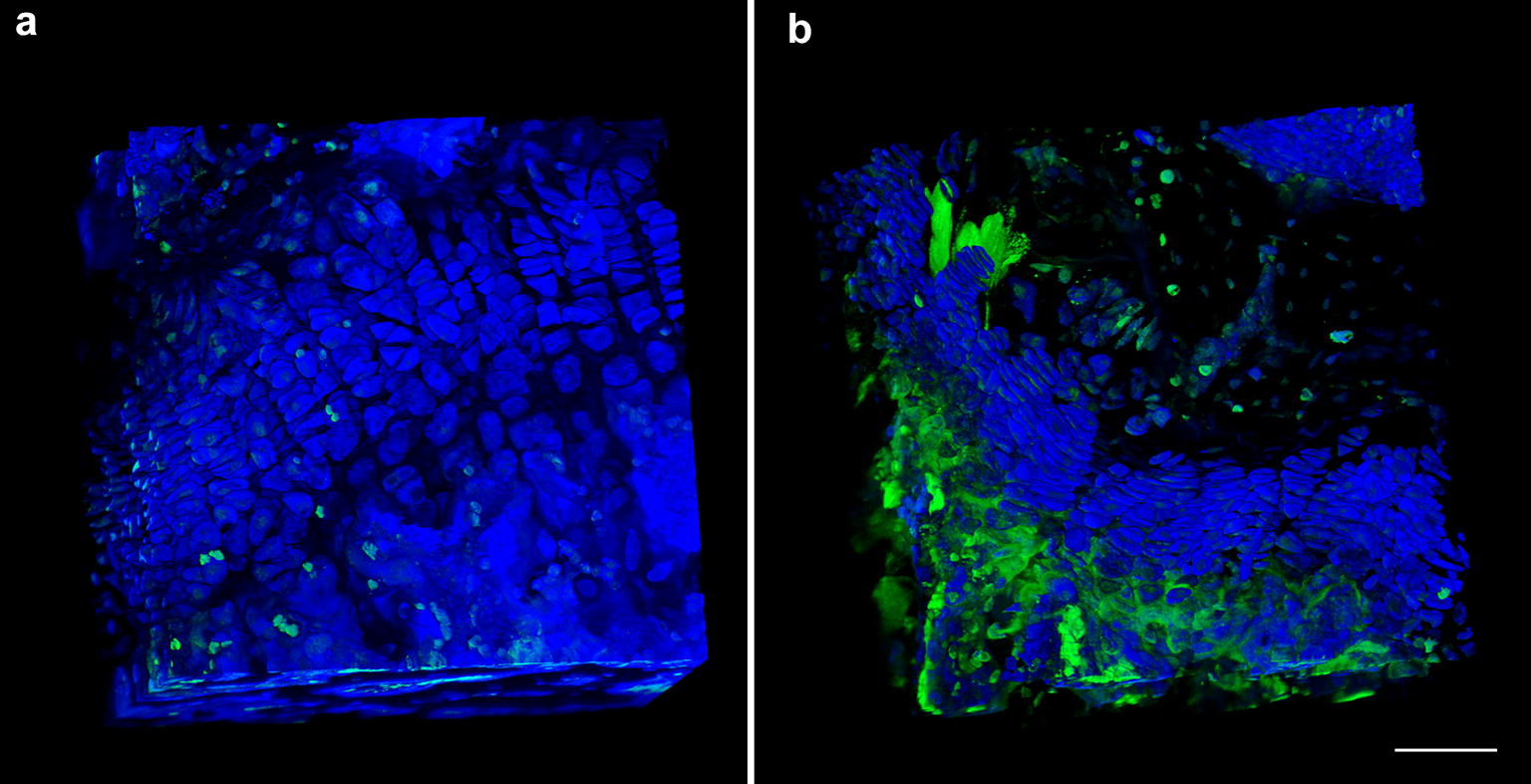
Confocal scans of growth plate cartilage in **a** lean and **b** obese mice. Chondrocytes in the growth plates of ob/ob mice do not form linear columns while chondrocytes in growth plate cartilage of lean mice do. DAPI nuclear stain (blue). Scale bar is 25 µm

**Fig. 3:**
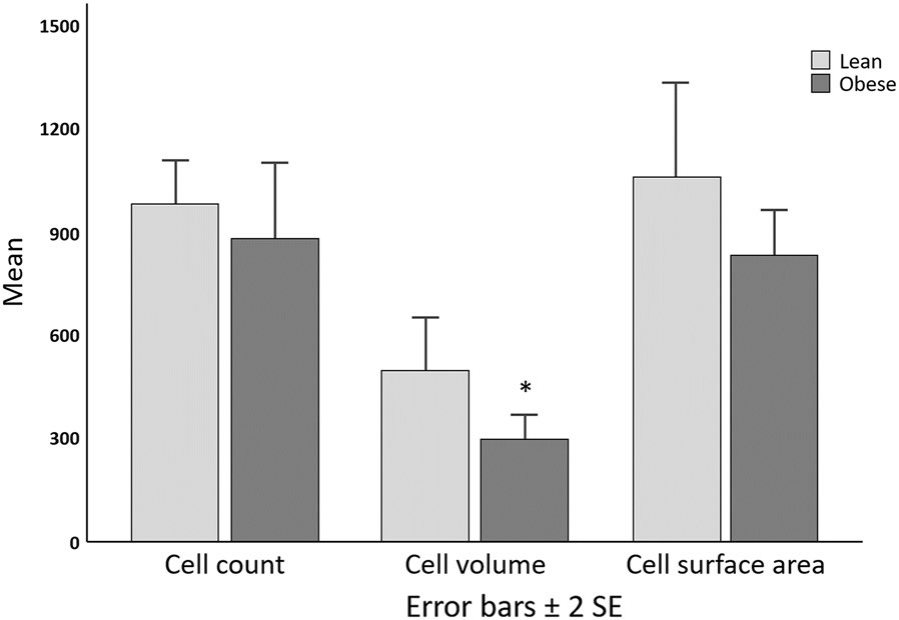
Chondrocyte count, volume (µm^3^) and surface area (µm^2^) in the proximal tibia growth plate in lean and ob/ob mice. *Cell volumes were significantly greater in lean mice in comparison to ob/ob mice (*P* < 0.05)

Chondrocytes in the proximal tibia growth plate were arranged into fewer cell columns in obese mice relative to lean mice (Table 1, *P* < 0.05). Columns of proliferating and hypertrophying chondrocytes were linearly arranged in the growth plates of lean mice (Figs. 1a, 2a). In obese mice, cell columns were shorter and less organized, particularly the hypertrophying cells in the distal portion of the growth plate (Figs. 1b, 2b). Chondrocyte volume was significantly reduced in obese mice in comparison to lean controls (*P* < 0.05), yet columns contain similar cell counts (Fig. 2, *P* > 0.05). Mean chondrocyte surface area was 24.1% greater in lean mice relative to their obese counterparts, although this difference was not statistically significant (Fig. 3, P > 0.05). Videos displaying the growth plate Z stacks of lean and obese mice being rotated along the x, y, and z axes are shown in Additional files 1 and 2.

## Discussion

Ob/ob mice are leptin deficient and exhibit metabolic profiles similar to obese T2DM patients. However, while ob/ob mice are unable to produce leptin, obese T2DM patients are often hyperleptinemic but demonstrate leptin resistance with similar metabolic consequences as leptin deficiency in ob/ob mice [15–18]. One of these consequences is reduced long bone mass. Like obese T2DM humans, ob/ob mice have a greater risk of bone fracture, along with significantly shorter long bones [9, 11, 19, 20]. Here, we report on growth plate structure in the tibias of ob/ob mice to assess structural differences that may correlate with reduced bone length.

**Fig. 4:**
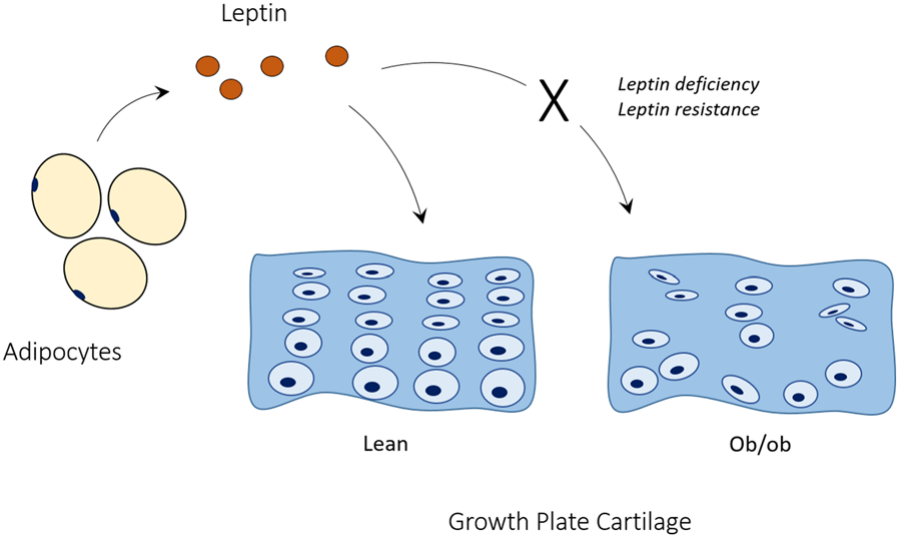
Schematic of the effects of leptin and leptin deficiency on growth plate cartilage of the tibia. Adipocyte-derived leptin regulates chondrocyte proliferation and arrangement in the growth plate. Lean mice demonstrate normal cartilage tissue microstructure while chondrocytes of leptin-deficient ob/ob mice have fewer columns of chondrocytes, with cells that are reduced in volume and have a more disorganized arrangement

As expected, ob/ob mice had greater body mass than lean controls. Consistent with prior reports [9–11], we also found ob/ob mice had shorter tibias than lean mice. Tibial longitudinal growth requires cartilage proliferation at the epiphyseal growth plate. Our analysis of growth plate cartilage three-dimensional structure showed obese mice have reduced growth plate height and fewer chondrocyte cell columns with cells that are reduced in volume. Cellular 3D arrangement is also more disorganized in growth plates of obese mice. These differences may be attributed to the inhibitory effects of leptin deficiency on chondrocyte metabolism (Fig. 4). Tibial growth plate chondrocytes of leptin-deficient mice exhibit disturbed 2D columnar structure with inhibited proliferation and extracellular matrix synthesis that are reversed when the cells are treated with leptin [21]. Similarly, patients with obese T2DM exhibit impaired chondrocyte proliferation and hypertrophy, as well as reduced collagen and proteoglycan expression [22–25]. Stiffness of the collagen network via crosslinking with advanced glycation end products also affects cartilage structure [26]. Altered cartilage extracellular composition in the diabetic state has been reported to affect mechanical properties of the tissue, thereby increasing the risk of tissue damage and impaired function [27–29]. This may also explain why obese patients with T2DM are at greater risk for osteoarthritis [30]. Susceptibility of chondrocytes to injury may also be a contributing factor to growth plate structural differences that inhibit growth. Thus, differences in tibial growth plate morphology of lean and ob/ob mice are similar to those observed in obese T2DM patients and may have origins that are multifactorial.

It should be noted that T2DM is known to affect the sexes differently. For example, females with T2DM are more likely to develop myocardial dysfunction and die from heart failure than males [31]. Females also respond more slowly and less robustly to the administration of exogenous leptin and insulin [32]. While we previously found reduced growth plate cartilage thickness and area in male and female mice [10, 13], it is unclear if three-dimensional microstructure differences between the sexes exist. Based on our previous findings, we hypothesize that growth plates of male ob/ob mice will exhibit similar differences in growth plate microstructure as lean controls, but further study is needed to evaluate this hypothesis. Additionally, serum leptin declines more quickly in females than males as they age, independent of body mass index and age-related endocrine changes [33]. There may be sex-related differences in the long-term implications of leptin resistance in children and adolescents with T2DM. Again, further study is needed to validate this prediction.

## Conclusions

Our data are in agreement with the mounting evidence that leptin deficiency has a significant impact on the length of long bones in ob/ob mice by affecting growth plate cartilage [9–11, 21]. Our study supports the hypothesis that growth plate morphology in long bones is altered in obese, leptin-deficient mice. Our findings indicate growth plate cartilage exhibits atypical morphology with reduced cell volume and numbers of proliferating cell columns. Given the rise of obesity and T2DM in juveniles, further study is needed to elucidate the clinical implications of leptin dysregulation on long-term bone and joint health.

## Additional files

**Additional file 1.** Video of composite Z stacks of the tibial growth plate of a lean mouse rotated about the x, y, and z-axes. Z stacks were captured at 0.2 µm intervals over a range of 80–100 µm using ACS APO 40×/1.15 oil.

**Additional file 2.** Video of composite Z stacks of the tibial growth plate of an obese (ob/ob) mouse rotated about the x, y, and z-axes. Z stacks were captured at 0.2 µm intervals over a range of 80–100 µm using ACS APO 40×/1.15 oil.
